# Dietary patterns during pregnancy and health-related quality of life: The Japan Environment and Children’s Study

**DOI:** 10.1371/journal.pone.0236330

**Published:** 2020-07-27

**Authors:** Kayoko Miura, Ayako Takamori, Kei Hamazaki, Akiko Tsuchida, Tomomi Tanaka, Hideki Origasa, Hidekuni Inadera, the Japan Environment and Children’s Study Group

**Affiliations:** 1 Toyama Regional Center for JECS, University of Toyama, Toyama, Japan; 2 Department of Public Health, Faculty of Medicine, University of Toyama, Toyama, Japan; 3 Department of Pediatrics, Faculty of Medicine, University of Toyama, Toyama, Japan; 4 Department of Biostatistics and Clinical Epidemiology, Faculty of Medicine, University of Toyama, Toyama, Japan; Ehime University Graduate School of Medicine, JAPAN

## Abstract

**Background:**

Limited research exists on how dietary pattern (DP) influences pregnant women’s health-related quality of life (HRQOL). This study aimed to identify DPs in a cohort of 92,448 pregnant Japanese women using fixed data from the Japan Environment and Children’s Study (JECS) to investigate the associations of DP with HRQOL.

**Methods:**

During the first trimester, DPs were assessed using the Food Frequency Questionnaire (FFQ), and HRQOL was assessed using the 8-Item Short-Form Health Survey (SF-8). DPs such as Western, Japanese and Unbalanced DP were identified through principal component analysis (PCA). Multivariable logistic model analysis was used to assess the associations between DP and HRQOL as good or poor.

**Results:**

We found a significant association between poor mental HRQOL in the univariate analysis for the Western DP (p = 0.014). However, this association was not significant in the multivariate analysis adjusted for basic confounders (p = 0.078). Western DP was not highly associated with poor physical HRQOL (from low-medium to high levels of intake; adjusted odds ratio [OR] 0.87–0.88, all p≤0.001, when comparing highest to lowest intake levels). A high intake of the Japanese DP was significantly associated with poor mental HRQOL and physical HRQOL (adjusted OR 1.20, p<0.0001 and adjusted OR 1.12, p = 0.005, respectively). A medium-high intake of the Unbalanced DP was not highly associated with poor physical HRQOL (adjusted OR 0.93, p = 0.048) but with poor mental HRQOL (adjusted OR 1.29, p<0.0001).

**Conclusion:**

This is the first known prospective study to establish an association between DP and HRQOL in pregnant women. We hope that our findings will help in the field of nutritional science.

## Introduction

Studies have reported an association between dietary pattern (DP) in pregnant women and their mental health, but these findings are somewhat controversial. Okubo et al. [1] reported no association between specific DP and postpartum depression risk. In contrast, Chatzi et al. [2] suggested that a healthy DP with regular intake of vegetables, fruits, beans, nuts, dairy products, and olive oil reduced the risk of postpartum depression. Additionally, Baskin et al. [3] observed that both prenatal and postpartum depression were related to DP during pregnancy, and an unhealthy DP (characterized by an intake of candy, grains, high-energy beverages, and fast foods) was associated with depressive symptoms during late pregnancy. Moreover, although studies on expectant mothers’ mental health typically focus on postpartum depression, in many cases, depressive symptoms develop before or during pregnancy. Thus, through cross-sectional analysis, it is important to investigate possible associations between expectant mothers’ DP during pregnancy and mental health. Furthermore, these associations should be studied according to country-specific DP due to varied diets between countries. Japan has a unique traditional diet influenced by its status as an island separate from mainland Asia. Although the modern eating habits of the Japanese population are becoming increasingly westernized, their DP are still considered to be strongly impacted by local cultural and social influences. However, as the physiological need for nutrition remains the same regardless geographical location, this study’s findings will provide important information for pregnant women around the world.

Health-related quality of life (HRQOL) is an emerging index increasingly used worldwide to measure an individual’s subjective evaluation of overall, self-reported health and well-being. The Seguimiento University of Navarra (SUN) cohort study investigated associations between diet and diseases such as hypertension, diabetes, obesity, and coronary heart disease, and their findings indicated an association between DP and HRQOL [4]. According to the SUN study, a Mediterranean DP featuring an intake of vegetables, fruits, cereals, legumes, and fish showed an association with physical HRQOL (Physical Component Summary; PCS). PCS is composed of four scales assessing physical function, role limitations caused by physical problems, bodily pain, and general health. Higher scores represent subjective recognition better physical health.

This study aimed to identify DP before and during the first trimester based on the Food Frequency Questionnaire (FFQ) for pregnant women from the Japan Environment and Children’s Study (JECS) data. Second, it aimed to clarify the association between DP and mental/physical HRQOL.

## Methods

### Study population

This study was based on 104,102 cases concerning parturition recorded, including 103,099 pregnancies recruited in the JECS. The JECS is a nationwide, multicenter, prospective birth cohort study conducted by the Ministry of the Environment of Japan; details on the study design have been previously reported [5, 6]. The aim of the JECS is to evaluate the effects of various environmental factors on children’s health and development.

A total of 15 catchment areas in Japan were selected to recruit pregnant women for the JECS between January 2011 and March 2014 [5, 6]. The JECS data [7, 8] are primarily based on self-administered questionnaires that include items such as medical treatment, age, weight, smoking history, lifestyle, occupation, demographic factors, and socioeconomic status. The SF-8 questionnaire (used to measure HRQOL) and the FFQ (used to measure dietary habits) were jointly named the MT1 questionnaire, which investigated pre-pregnancy status and were completed by expectant mothers in their first trimester.

This study was based on both the jecs-ag-20160424 dataset distributed to interested researchers in June 2016 and the allbirth_revice001_ver001 data set distributed in October 2016.

**Fig 1:**
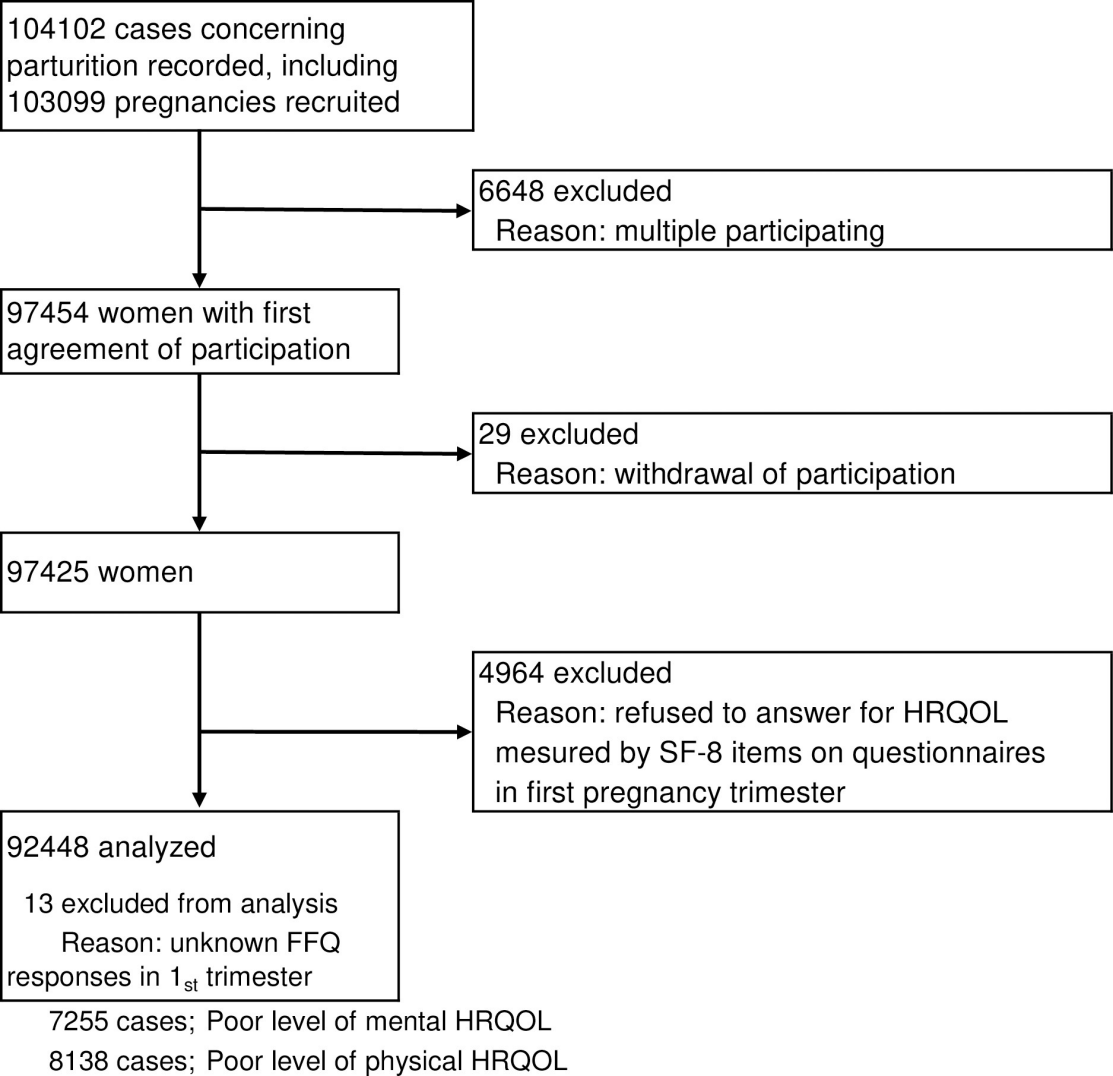
Flow diagram of the recruited and excluded pregnant women participating in the Japan Environment and Children's Study (JECS) to evaluate the association between health-related quality of life (HRQOL) and dietary pattern (DP).

Data from 92,448 women were included in the present analyses after excluding 6,648 multiple participation cases, 29 withdrawal of participation cases, 4,964 unknown SF-8 responses, and 13 unknown FFQ responses (Fig 1).

### Health-related quality of life

The outcome measured in this study was HRQOL, using HRQOL instruments that provide a useful means of measuring health outcomes at the population level. The SF-8 questionnaire was used to measure HRQOL and selected for its brevity (1–2 min administration time) [9]. The SF-8 instrument, which provides a generic measure of physical and mental health status that is not specific to age or disease, comprises the following eight items, each representing one health domain: 1) general health perceptions, 2) physical functioning, 3) role limitations due to physical health problems, 4) bodily pain, 5) vitality (energy/fatigue), 6) social functioning 7) mental health, and 8) role limitations due to emotional problems. These items were investigated through the following questions:

1. Overall, how would you rate your health during the last four weeks?
2. How much did physical health problems limit your usual physical activities (such as walking or climbing stairs) in the last four weeks?
3. During the last four weeks, how much difficulty did you face doing your daily work, both at home and away from home, because of your physical health?
4. During the last four weeks, how much bodily pain have you had?
5. During the last four weeks, how much energy did you have?
6. During the last four weeks, how much did your physical health or emotional problems limit your usual social activities with family or friends?
7. During the last four weeks, how much have you been bothered by emotional problems (such as feeling anxious, depressed, or irritable)?
8. During the last four weeks, how much did personal or emotional problems keep you from doing your usual work, school, or other daily activities?

Overall HRQOL is indicated by two summary assessment scores: the first four health domains generate the physical component summary (PCS) score, and the last four reflect psychological features and generate the mental component summary (MCS) score. Higher scores on the PCS and MCS correspond to better health status. In this study, we investigated and defined 70 as the maximum summary score; accordingly, half of the score 35 was considered the cutoff point for PCS and MCS assessments. A summary score of ≤35 indicates poor HRQOL.

### Dietary information

Dietary information data were obtained from self-reported questionnaires during the first trimester. The questionnaires were administered in the stable period of pregnancy and investigated dietary habits before and during pregnancy (approximately 12 months), with questions concerning pre- or early pregnancy. The FFQ used in the JECS was a validated, self-administered diet questionnaire that has been evaluated in previous studies with respect to nutrient factors [10, 11]. The FFQ has been used to assess subjects’ nutrient and food intake in large-scale epidemiologic studies from several countries including Japan [12–14]. The FFQ included food lists (cereals, eggs, fat and oils, fish and shellfish, fruits, meats, mushrooms, milks, potatoes and starches, and vegetables), standard units (volumes; 0.5 for small, 1.0 for medium, and 1.5 for large), portion sizes (e.g., 30 g portion−1), and frequencies (ranging from less than once per month to more than seven times per day). Moreover, eating habits (e.g., frequency of having breakfast, eating out, and eating speed) were assessed using the questionnaire. The subjects reported their intake frequency and portions of consumption in the past year. Dietary intake was calculated using the frequencies, standard units, and portion sizes for the various food items of the FFQ. This amount of intake was subsequently energy adjusted using the energy density method [15]. To investigate relationships between DP and HRQOL, the FFQ structured a list of 167 food and beverage items that were used in our study from the JECS dataset. Based on these lists, food intake variables were calculated as 23 major food groups including detailed food types.

### Statistical analysis

To identify the pregnant women’s DP, we first applied principal component analysis (PCA) to FFQ-related variables in major groups and in detailed groups, not including foods with small intake such as alcohol, water, and seasonings. Eigenvectors obtained from the calculated covariance matrices revealed important patterns in the FFQ data that allowed us to interpret various food groups as major DPs.

Second, to evaluate associations between levels of intake for each major DP and PCS and MCS, we applied univariate and multivariate logistic regression analyses. Good and poor HRQOL were used as outcomes. Intake levels were categorized according to quartiles (quartile 1, low intake; quartile 2, low-medium; quartile 3, medium-high; quartile 4, high). The following potential confounders were considered: mother age, body mass index (BMI) (kg/m^2^) before pregnancy, number of previous pregnancies (0, meaning first birth/1/2/3+), spontaneous abortion history, induced abortion history, artificial insemination, abnormal pregnancy complications, medication, hypertension, hyperlipidemia, anemia, diabetes mellitus, neurological/mental diseases, digestive diseases, occupation, educational background and family income, smoking history, alcohol history, and energy.

Odds ratios (ORs) and 95% confidence intervals (CIs) were analyzed. Two-sided p-values <0.05 were considered statistically significant. SAS statistical software version 9.4 (SAS Institute Inc., Cary, NC) was used for all analyses.

### Ethics

This study was conducted according to the guidelines established in the Declaration of Helsinki, and all procedures involving human subjects/patients were approved by the Ethics Committee of the University of Toyama (No. 28–75). Written informed consent was obtained from all participating women and their partners.

## Results

Of the total 92,448 pregnant women analyzed in this study, 7255 (7.85%) and 8138 (8.80%) had poor mental HRQOL and physical HRQOL, respectively, (Fig 1 and Table 1). Approximately 5% of the total study population reported scores ≤35.

**Table 1:** Characteristics of pregnant women with poor mental and physical HRQOL measured by SF-8.

	Poor mental HRQOL^†^	Poor physical HRQOL^‡^	All
	(n = 7255)	(n = 8138)	(n = 92448)
Mother age (year), *mean (SD)*	30.30 (5.11)	31.44 (4.75)	30.80 (5.05)
BMI before pregnancy (kg/m^2^), *mean (SD)*	21.12 (3.30)	21.13 (3.25)	21.22 (3.28)
Number of previous pregnancies, *n (%)*			
0	2416 (33.58)	2598 (32.16)	29399 (32.07)
1	2245 (31.20)	2687 (33.26)	30300 (33.05)
2	1401 (19.47)	1598 (19.78)	18304 (19.97)
3+	1133 (15.75)	1196 (14.80)	13666 (14.91)
Spontaneous abortion history	1426 (19.66)	1805 (22.18)	18324 (19.82)
Induced abortion history	1316 (18.14)	1166 (14.33)	13638 (14.75)
Artificial insemination	147 (2.03)	260 (3.19)	2383 (2.58)
Abnormal pregnancy complications	406 (5.60)	530 (6.51)	4955 (5.36)
Medication	2796 (38.54)	3451 (42.41)	30036 (32.49)
Past medical history			
hypertension	50 (0.69)	43 (0.53)	436 (0.47)
hyperlipidemia	43 (0.59)	40 (0.49)	463 (0.50)
anemia	1557 (21.46)	1744 (21.43)	17032 (18.42)
diabetes mellitus	72 (0.99)	87 (1.07)	821 (0.89)
neurological/mental diseases	1108 (15.27)	962 (11.82)	7312 (7.91)
digestive diseases	1275 (17.57)	1527 (18.76)	11781 (12.74)
Occupation, *n (%)*			
full time	2214 (31.52)	2631 (33.44)	30446 (34.03)
part time	1810 (25.77)	1835 (23.33)	23720 (26.51)
homemaker	3000 (42.71)	3401 (43.23)	35293 (39.45)
Educational background, *n (%)*			
Junior high- / high-school, or technical-college	2926 (41.64)	2667 (33.69)	33736 (37.51)
vocational college	1614 (22.97)	1828 (23.09)	20562 (22.86)
Junior college	1146 (16.31)	1439 (18.18)	15904 (17.69)
University or higher educational institution	1341 (19.08)	1983 (25.05)	19726 (21.94)
Family income (yen/year), *n (%)*			
< 4,000,000 JPY	2953 (45.04)	2801 (37.34)	33573 (39.93)
4,000,000–5,999,999 JPY	2071 (31.58)	2543 (33.90)	27818 (33.08)
6,000,000–7,999,999 JPY	931 (14.20)	1278 (17.04)	13480 (16.03)
≥ 8,000,000 JPY	602 (9.18)	879 (11.72)	9211 (10.95)
Smoking history			
never	3826 (53.12)	4971 (61.39)	53596 (58.36)
former	2935 (40.75)	2911 (35.95)	33867 (36.88)
current	441 (6.12)	215 (2.66)	4371 (4.76)
Alcohol history			
never	2518 (34.87)	2939 (36.29)	31685 (34.42)
former	4030 (55.8)	4365 (53.90)	51079 (55.49)
current	674 (9.33)	794 (9.80)	9287 (10.09)
Energy (kcal), mean (SD)	1915.33 (897.30)	1857.24 (773.24)	1829.57 (822.69)

*Abbreviations*: HRQOL, health related quality of life; BMI, Body Mass Index

^†^ Defined from the SF-8 mental component summary (MCS) score points < 35 (*see* *method* *section*)

^‡^ Defined from the SF-8 physical component summary (PCS) score points < 35 (*see* *method* *section*)

### Dietary patterns

Table 2 shows the PCA-derived factor loading matrices for the intake of each food. Three DPs were identified as follows: the Western DP characterized by the intake of grain cereals, vegetables, fruits, milk, and yogurt; the Japanese DP characterized by the intake of foods such as rice, miso soup, Japanese wheat noodles, grain cereals, beans, vegetables, fruits, meats, Japanese tea, vegetable juice, and 100% fruit juice, and also by low intake of milk; and the Unbalanced DP characterized by a high intake of grain cereals, bottled tea, bottled coffee, and carbonated drinks, and low intake of green and yellow vegetables, fruits, and green tea.

**Table 2:** Eigenvectors of various food groups in the three major dietary patterns (DP) by applying principal component analysis.

	Eigenvectors^†^		
	*Factor 1*	*Factor 2*	*Factor 3*
Food groups	Western DP	Japanese DP	Unbalanced DP
detailed types of foods			
Rice	- ^‡^	**0.116**	0.015
Miso soup	0.014	0.061	-0.048
Low-fat milk	-	-	-
Whole milk	**0.138**	-0.021	0.012
Eggs	**0.484**	**-0.190**	0.028
Yogurt	0.033	0.016	-
Bread	**0.147**	-	-0.028
Udon^a^	0.026	0.028	-
Buckwheat noodles	0.017	**0.079**	0.013
Chinese noodles	-	0.032	-
Pasta	-	0.046	0.019
Somen and Hiyamugi^a^	-	0.048	-
Ice cream	-	0.043	-
Tohu	0.011	0.023	-
Natto^b^	-	0.028	-
Potatoes	-	0.020	-
Green tea (Infusion)	-	0.017	-
Green tea (in cans and bottles)	0.018	**0.097**	**-0.166**
Oolong tea (Infusion)	-	**0.180**	**0.799**
Oolong tea (in cans and bottles)	-	0.033	0.031
Black tea (Infusion)	0.011	**0.123**	**0.405**
Black tea (in cans and bottles)	-	0.028	-
Coffee (Infusion)	-	**0.057**	**0.078**
Coffee (Instant)	-	0.020	-
Coffee (in cans and bottles)	0.021	0.025	-
Vegetable juice	0.013	**0.052**	**0.087**
Orange juice	0.020	**0.089**	-0.031
Apple juice	0.017	**0.100**	-
Grapefruit juice	0.015	**0.088**	-
Drink 100% fruit juice	0.012	**0.071**	-
Drink not 100% fruit juice	-	**0.057**	0.030
Carbonated drink	0.023	**0.170**	**0.204**
Soymilk	0.012	0.039	-
Lactic acid bacteria beverage	0.035	0.043	0.011
Other soup	0.015	**0.084**	0.020
major types of foods			
Cereals	**0.082**	**0.408**	**0.057**
Potatoes and starches	0.010	0.041	-0.014
Beans	0.039	**0.129**	-0.046
Vegetables	**0.107**	**0.444**	**-0.226**
Japanese pickles	-	0.035	-0.013
Green and yellow vegetables	**0.060**	**0.244**	**-0.114**
Other vegetables	0.047	**0.200**	**-0.111**
Fruits	**0.114**	**0.529**	**-0.150**
Seafood	0.016	0.048	-
Meats	0.025	**0.077**	0.016
Eggs	0.033	0.016	-
Milk	**0.823**	**-0.131**	0.029
Confectioneries	0.011	0.037	-
Variation explained (%)	32.5	11.6	6.8
Correlation with energy intake, coefficient	0.6	0.6	<0.1

^a:^ Japanese wheat noodle

^b:^ fermented soybeans as abbreviations.

^†^ Absolute values > 0.05 are indicated in bold.

^‡^ Absolute values <0.01 are indicated using ‘-’.

The energy intake was correlated with DP (correlation coefficients with Western DP and Japanese DP were 0.6, respectively, Table 2). To avoid statistical multicollinearity issues, we did not include energy intake as a covariate in the multiple logistic regression model (Table 3).

**Table 3:** Association of dietary pattern with poor level of HRQOL for subjective mental health (mental HRQOL) and subjective physical health (physical HRQOL) by applying logistic regression analyses.

Dietary Patterns	Quartile	unadjusted OR (95%CI)	P-value	adjusted OR (95%CI)	P-value
A) poor mental HRQOL					
Western DP	1 (Low)	reference		reference	
	2 (Low-medium)	0.95 (0.89, 1.02)	0.152	0.97 (0.90, 1.05)	0.476
	3 (Medium-high)	0.92 (0.86, 0.98)	0.014	0.93 (0.86, 1.01)	0.078
	4 (High)	1.01 (0.95, 1.08)	0.683	1.02 (0.95, 1.10)	0.574
Japanese DP	1 (Low)	reference		reference	
	2 (Low-medium)	1.00 (0.93, 1.07)	0.902	1.02 (0.94, 1.10)	0.683
	3 (Medium-high)	0.97 (0.90, 1.04)	0.330	0.99 (0.92, 1.07)	0.812
	4 (High)	1.20 (1.12, 1.28)	< .0001	1.20 (1.11, 1.30)	< .0001
Unbalanced DP	1 (Low)	reference		reference	
	2 (Low-medium)	0.95 (0.88, 1.02)	0.124	0.98 (0.90, 1.06)	0.589
	3 (Medium-high)	1.07 (1.00, 1.15)	0.044	1.08 (1.00, 1.17)	0.065
	4 (High)	1.33 (1.25, 1.42)	< .0001	1.29 (1.20, 1.40)	< .0001
B) poor physical HRQOL					
Western DP	1 (Low)	reference		reference	
	2 (Low-medium)	0.96 (0.90, 1.03)	0.249	0.88 (0.82, 0.95)	0.001
	3 (Medium-high)	0.99 (0.93, 1.05)	0.682	0.87 (0.81, 0.93)	0.0001
	4 (High)	1.01 (0.95, 1.08)	0.684	0.88 (0.82, 0.95)	0.001
Japanese DP	1 (Low)	reference		reference	
	2 (Low-medium)	1.07 (1.00, 1.14)	0.056	1.02 (0.95, 1.10)	0.551
	3 (Medium-high)	1.11 (1.04, 1.19)	0.001	1.09 (1.01, 1.17)	0.020
	4 (High)	1.16 (1.08, 1.23)	< .0001	1.12 (1.03, 1.20)	0.005
Unbalanced DP	1 (Low)	reference		reference	
	2 (Low-medium)	0.85 (0.80, 0.90)	< .0001	0.89 (0.83, 0.95)	0.001
	3 (Medium-high)	0.83 (0.78, 0.88)	< .0001	0.93 (0.86, 1.00)	0.048
	4 (High)	0.85 (0.79, 0.90)	< .0001	0.97 (0.90, 1.04)	0.351

Abbreviations: DP, dietary pattern; HRQOL, health related quality of life; OR, odds ratio; 95%CI, 95% confidence interval

Adjusted odds ratio (OR) was estimated from multivariate logistic regression model considering following confounders; mother age, BMI before pregnancy, number of previous pregnancies, spontaneous abortion history, induced abortion history, artificial insemination, abnormal pregnancy complications, medication, hypertension, hyperlipidemia, anemia, diabetes mellitus, neurological/mental diseases, digestive diseases, occupation, educational background, family income, smoking history, alcohol history as confounders (categories were shown in Table 1).

### Mental HRQOL

Table 3A shows the associations between the intake level of each DP and the risk of poor mental HRQOL. The first quartile (low intake group) was used as the reference for each DP. High intake of Japanese DP was significantly associated with poor mental HRQOL in both univariate (unadjusted OR 1.20, p<0.0001) and multivariate (adjusted OR 1.20, p<0.0001) analyses after adjusting for confounding factors. For the Unbalanced DP, pregnant women in the high intake quartiles showed a significantly increased risk of poor mental HRQOL (adjusted OR 1.29, p<0.0001). Only medium-high intake of the Western DP showed a relationship with poor mental HRQOL in the univariate analysis (p = 0.014); however, this association disappeared in the multivariate analysis after adjusting for confounders (p = 0.078).

### Physical HRQOL

Table 3B shows the associations between the intake level of DP and physical HRQOL. As with mental HRQOL, the first quartile (low food intake) was used as the reference. The low-medium to high intake quartiles of the Western DP showed a significant association with decreased risk of poor physical HRQOL in multivariate analysis after adjusting for confounders (adjusted OR for confounders ranged from 0.87–0.88, all p≤0.001), but this association was not shown in univariate analysis. For the Japanese DP, pregnant women in medium-high and high intake groups showed a significant association with an increased risk of poor physical HRQOL (adjusted OR 1.09 and 1.12, p = 0.020 and 0.005, respectively). This was observed in both univariate and multivariate analyses. For the Unbalanced DP, pregnant women in low-medium and medium-high intake groups exhibited significant associations with good physical HRQOL (adjusted OR 0.89 and 0.93, p = 0.001 and 0.048, respectively). The high intake group of the Unbalanced DP was not positively associated with physical HRQOL after adjusting for confounders (p = 0.351).

## Discussion

This study identified the DP in Japanese pregnant women and clarified associations between intake levels of these DPs and physical/mental HRQOL. Our findings revealed that other intake levels of the Western diet lowered the risk of poor physical HRQOL by 0.87–0.88 times when compared to low intake levels. This indicates that pregnant women in Japan who prefer eating Western foods feel physically healthy. This Western DP might usually indicate an unhealthy DP that is highly related to sugar-beverage and high fat intake, but we considered it to be a healthy-Western DP than unbalanced eating habits. Further, we found a significant association between participants’ mental HRQOL in the univariate analysis for the third quartile of adherence to this Western DP. However, this association was not significant in the multivariate analysis adjusted for basic confounders. From these results, we could conclude that the association between Western DP and mental HRQOL was an untrue relationship influenced by confounders.

Further, our data indicated that pregnant women with high adherence to the Japanese DP felt that they had poor mental and physical health. Pregnant women with a high adherence to Japanese DP had a 1.20 higher risk of a poorer mental HRQOL than those who had low adherence to Japanese DP, and this association was significant in both univariate and multivariate analyses. In addition, the 3^rd^ and 4^th^ quartiles of adherence to Japanese DP were associated with poorer physical HRQOL in both univariate and multivariate analyses. The groups with medium-high and high intake of Japanese foods faced the risk of poor physical HRQOL that was 1.09–1.12 times higher than that of the low intake group.

Similarly, women who reported the 4^th^ quartiles of adherence to the Unbalanced DP intake had a 1.29 times higher risk of poor mental HRQOL than those reporting low intake of an Unbalanced DP; this association was significant in both univariate and multivariate analyses. Interestingly, however, the physical HRQOL results for women in this DP were opposite to those for mental HRQOL. The low-medium and medium-high adherence to an Unbalanced DP intake was associated with good (non-poor) physical HRQOL, 0.89–0.93 times less than the low adherence. Thus, pregnant women with a tendency to consume an Unbalanced DP were more likely to report feeling physically healthier than those who did not consume this diet, which is the same association found for high adherence to Western DP.

As Japanese food has generally been considered healthier overall than Western food, the results of our study are surprising. A previous survey of the general adult population reported that Japanese and healthy DP were associated with a reduced risk of depression [16] and suicide [17]. From this perspective, our findings—that the intake of Japanese food generally results in a lower HRQOL—are unexpected. Furthermore, it is curious that both healthy Japanese DP and unhealthy Unbalanced DP show approximately the same associations and directions with poor mental HRQOL. There may be several reasons as to why high adherence to an Unbalanced DP could for instance be associated with good physical health. As the causal relationship was not clear in our study, it may have been possible that physical fatigue required an adherence to Japanese DP and intake was introduced. As pregnant women with Unbalanced DP were physically feeling good, they exercised and were likely to be eating whatever, wherever, and in whatever amounts they wanted, without stress.

It has been suggested that the mental and physical HRQOL of pregnant women who consumed excessive Japanese food, as in the high intake group, was worse than that of pregnant women who did not. There are some possible explanations for this.

First, several studies have examined the association between HRQOL and the immune system [18–21]. It has been suggested that subjects with poor HRQOL may have increased levels of inflammatory biomarkers interleukin (IL)-1β, IL-6, tumor necrosis factor (TNF)-α, and C-reactive protein (CRP), compared to subjects with good HRQOL. Pregnant women with immune function that is typically worse than the general population have shown an association between poor HRQOL and IL-6 [21]. Additionally, some foods have been shown to promote inflammation, leading to worse immune function. Shivappa et al. [22] examined the effect of 45 types of nutrients and foods on six inflammatory biomarkers (IL-1β, IL-4, IL-6, IL-10, TNF-α, and CRP) and established the “dietary inflammatory index (DII)” scoring method, and the results reported that saturated fatty acids, carbohydrates, and vitamin B12 were found to promote inflammation. The Japanese diet is high in vitamin B12, which is found in fish, shellfish, poultry liver, marine algae, and soybeans. Furthermore, carbohydrates are the main food group of the Japanese diet. Therefore, pregnant women with a high intake of the Japanese diet are ingesting foods with a high proinflammatory function, and the combined effects of these nutrients might lower their immune function, which could consequently lower their HRQOL.

To confirm whether the Japanese DP in this study was high in foods with high DII scores, we evaluated the nutrients consumed by pregnant women with a high intake of the Japanese diet. We observed that pregnant women in the 4^th^ quartile of adherence to the Japanese DP consumed a higher level of these nutrients than those in the 1^st^ quartile. Among women with poor mental HRQOL, those with the 1^st^ quartile of adherence to the Japanese DP consumed 184.8 g carbohydrates, 3.8 μg vitamin B12, and 19.5 g saturated fatty acids per day. In contrast, those with a high intake of the Japanese diet consumed 353.1 g carbohydrates, 6.7 μg vitamin B12, and 26.3 g saturated fatty acids (S1 Table). Among women with poor physical health, those with a low intake of the Japanese DP consumed 184.4 g carbohydrates, 3.7 μg vitamin B12, and 19.2 g saturated fatty acids, whereas those with a high intake consumed 337.3 g carbohydrates, 6.2 μg vitamin B12, and 24.5 g saturated fatty acids (S1 Table). As associations between foods with proinflammatory action and depression or psychological distress have been suggested in previous studies [23–25], similar associations might be found in pregnant women with poor mental or physical HRQOL whose diet is comprised of a high percentage of proinflammatory foods. However, future studies are needed to clarify these possible mechanisms.

It may be possible that individuals with high health consciousness could have poorer HRQOL, although this seems paradoxical. In the Japanese DP identified in this study, the number of items that were actively consumed by pregnant women was higher than the number of foods consumed in the other two DP. Accordingly, this pattern is characterized by a balanced and diverse food intake. Women who prefer a balanced diet, and thus seem to have a high awareness of or higher commitment to developing healthy eating habits, might have poorer self-rated health. Orthorexia, a term first used by Steven Bratman in 1997, refers to a state where an individual becomes obsessed with eating a “healthy” diet [26]. Although orthorexia is thought to be a type of eating disorder and research on the condition is increasing [27], it is not described in the Diagnostic and Statistical Manual of Mental Disorders; therefore, no clear definition or diagnostic criteria exist. It is possible that Japanese food itself does not negatively affect HRQOL, but rather that women inclined towards orthorexia may be more likely to consume a traditional Japanese diet. It could also be possible that women with existing health problems (and therefore poorer HRQOL) might be more likely to try and eat a balanced diet like meat, vegetables, fruits, etc., in an effort to alleviate their other health-related symptoms. Japanese DP was characterized by a high intake of Somen noodles, coffee, and fruit juices and a low intake of dairy products. In Japan, Somen is considered quick and easy to cook, and women often consume fruit juice when they are tired. The Western DP had adherence to dairy product intake. The animal protein contained in milk is considered to help muscle or bodily function. However, lactase is necessary for the digestion of milk to break down lactose, but it is known that few Japanese people are able to produce this enzyme. This point might support the association between Western DP and good physical HRQOL. In the Unbalanced DP, the number of foods that were actively consumed by pregnant women was the lowest among the three DPs; conversely, the number of foods that women were reluctant to consume was highest. Therefore, it is likely that the health consciousness of women in this group was rather low, and that their likes and dislikes of food were clear.

To the best of our knowledge, this study is the first to examine associations between DP and HRQOL in Japanese pregnant women, and offers important insights. First, we conducted an analysis based on accurate meal-related information collected in a large-scale cohort survey of approximately 100,000 pregnant women. Due to the large sample size used in this study—larger than the previous comparable studies, the DP and findings of this study may be more generalizable for other populations. Second, we performed multivariate analyses adjusted for confounding factors, which enabled us to examine the association between DP and self-rated health by adjusting for confounders that might affect HRQOL. Finally, as this study was based on data obtained by the JECS, which includes data of approximately 100,000 individuals in 15 regions throughout Japan, the possibility of selection bias is likely low. However, this study also has some limitations. First, we could not establish causality due to the cross-sectional design of this study. Second, as the survey used self-administered questionnaires, recall bias and individual comprehension of questionnaire items could affect the responses. Third, the FFQ we used has not been validated for use with pregnant women. Fourth, as the dietary survey collected information about women’s eating habits during the past year, answers might have reflected pre-pregnancy eating habits. As it is possible that dietary habits may change after pregnancy, we intend to examine this further in a future study. Some pregnant women might have had nausea and vomiting because our FFQ data were collected in the first trimester; however, this would not have much of an impact. This is because the FFQ was administered in the stable period of pregnancy and investigated dietary habits before and during pregnancy (approximately 12 months).

In conclusion, we identified three DPs—Western, Japanese, and Unbalanced—in Japanese pregnant women and clarified associations between intake levels of each diet and HRQOL. Women with a low-medium to high intake of the Western DP during pregnancy were significantly more likely to report good physical HRQOL compared to the low intake level group; however, no correlation was found with mental HRQOL. Thus, pregnant Japanese women with good HRQOL might be inclined to prefer a Western diet. Women with a medium-high intake of the Unbalanced DP reported good physical HRQOL and poorer mental HRQOL. Factors related to poor mental HRQOL, such as stress, might lead to a high intake of an Unbalanced diet. Finally, and surprisingly, a high intake of the Japanese DP was related to poorer physical HRQOL and poorer mental HRQOL when compared with a low intake level. This is the first known prospective study to establish an association between DP and HRQOL in pregnant women. In addition to focusing on abstinent diets that increase the risk of childbirth, there is the potential for advice from DP and nutrients to approach pregnant women’s subjective quality of life. We hope that our findings will help in the health care of pregnant women.

## Supporting information

S1 Table Characteristics of nutrients needed by pregnant women and DP. (DOCX)
