# Supplementary material for: Dietary patterns during pregnancy and health-related quality of life: The Japan Environment and Children’s Study
Source: PLoS One. 2020 Jul 27;15(7):e0236330. doi: 10.1371/journal.pone.0236330 (PMC7384865; doi:10.1371/journal.pone.0236330)
Supplement: Supplementary file 1 [file pone.0236330.s001.docx]

	Mental				Physical			
	**Western DP with non-poor mental HRQOL**				**Western DP with non-poor physical HRQOL**			
Nutrients, *mean* ± *SD*	1st quartile	2nd quartile	3rd quartile	4th quartile	1st quartile	2nd quartile	3rd quartile	4th quartile
Energy, kcal	1328.1 ± 419.9	1661.9 ± 461.3	1867.3 ± 539.4	2432.8 ± 1158.3	1327.4 ± 422.5	1664.3 ± 463.9	1873.9 ± 550.6	2443.3 ± 1177.2
Protein, g	42.3 ± 16.2	55.1 ± 17.7	63.7 ± 20.4	89.6 ± 48.7	42.3 ± 16.2	55.1 ± 17.8	63.9 ± 20.9	89.9 ± 49.4
Carbohydrates, g	188.7 ± 57.0	231.5 ± 64.4	254.8 ± 74.5	313.4 ± 145.5	188.7 ± 57.2	231.7 ± 64.6	255.6 ± 75.8	314.8 ± 148.0
Calcium, mg	236.8 ± 95.8	388.3 ± 101.3	533.6 ± 127.4	1089.9 ± 767.4	236.1 ± 95.9	388.2 ± 101.5	534.6 ± 128.5	1095.9 ± 772.8
Iron, mg	5.2 ± 2.0	6.6 ± 2.3	7.4 ± 2.8	9.2 ± 5.5	5.2 ± 2.0	6.6 ± 2.3	7.5 ± 2.8	9.2 ± 5.6
Vitamin B12, μg	2.8 ± 2.2	3.9 ± 2.7	4.7 ± 3.0	6.9 ± 6.8	2.8 ± 2.2	4.0 ± 2.7	4.8 ± 3.0	6.9 ± 7.0
Folic acid, μg	183.0 ± 88.1	250.3 ± 103.2	293.0 ± 126.8	391.6 ± 263.1	182.2 ± 87.3	249.7 ± 103.1	293.4 ± 127.0	392.4 ± 267.5
Saturated fatty acids, g	12.0 ± 6.6	16.2 ± 6.9	19.5 ± 8.0	31.0 ± 19.6	12.0 ± 6.6	16.2 ± 7.0	19.6 ± 8.2	31.1 ± 19.8
	**with poor mental HRQOL**				**with poor physical HRQOL**			
	1st quartile	2nd quartile	3rd quartile	4th quartile	1st quartile	2nd quartile	3rd quartile	4th quartile
Energy, kcal	1352.5 ± 448.5	1727.7 ± 512.2	1995.6 ± 673.9	2574.8 ± 1203.2	1357.5 ± 419.9	1696.4 ± 483.5	1908.0 ± 560.7	2454.9 ± 1004.0
Protein, g	43.1 ± 16.9	57.1 ± 19.3	68.4 ± 25.8	94.0 ± 49.4	43.9 ± 16.1	56.5 ± 18.3	65.3 ± 21.2	90.1 ± 41.4
Carbohydrates, g	190.1 ± 60.3	238.2 ± 69.7	268.8 ± 91.8	331.1 ± 159.6	190.1 ± 57.1	235.3 ± 66.6	258.6 ± 78.2	315.3 ± 133.2
Calcium, mg	240.8 ± 103.4	397.5 ± 108.2	556.5 ± 149.7	1146.2 ± 793.3	247.2 ± 101.3	397.2 ± 105.2	542.8 ± 137.7	1080.6 ± 738.1
Iron, mg	5.3 ± 2.2	6.9 ± 2.5	7.9 ± 3.2	9.7 ± 5.8	5.4 ± 2.2	6.8 ± 2.4	7.6 ± 3.0	9.4 ± 4.8
Vitamin B12, μg	2.9 ± 2.4	4.1 ± 2.8	5.1 ± 3.3	7.2 ± 7.1	3.0 ± 2.3	4.0 ± 2.5	4.8 ± 3.1	6.8 ± 4.7
Folic acid, μg	186.1 ± 93.1	255.9 ± 108.2	310.0 ± 146.0	413.4 ± 290.9	193.8 ± 99.4	261.2 ± 108.6	303.1 ± 141.7	403.7 ± 244.2
Saturated fatty acids, g	12.5 ± 6.8	17.3 ± 8.1	21.2 ± 10.1	32.9 ± 20.5	12.6 ± 6.7	16.6 ± 7.5	20.1 ± 8.0	31.3 ± 18.4
	**Japanese DP with non-poor mental HRQOL**				**Japanese DP with non-poor physical HRQOL**			
Nutrients, *mean* ± *SD*	1st quartile	2nd quartile	3rd quartile	4th quartile	1st quartile	2nd quartile	3rd quartile	4th quartile
Energy, kcal	1441.8 ± 716.1	1583.8 ± 430.2	1840.9 ± 469.6	2431.6 ± 1088.0	1442.3 ± 718.7	1584.1 ± 430.6	1843.3 ± 474.2	2444.5 ± 1107.8
Protein, g	51.9 ± 33.5	53.8 ± 19.7	62.3 ± 21.7	82.9 ± 44.6	51.9 ± 33.7	53.8 ± 19.6	62.4 ± 21.9	83.4 ± 45.3
Carbohydrates, g	183.8 ± 74.5	215.3 ± 46.6	253.1 ± 51.1	337.3 ± 137.4	183.9 ± 74.6	215.4 ± 46.9	253.4 ± 51.6	338.9 ± 139.9
Calcium, mg	598.0 ± 728.1	447.9 ± 316.9	505.1 ± 314.0	698.3 ± 522.1	599.6 ± 731.6	447.4 ± 316.6	505.6 ± 319.4	701.6 ± 526.4
Iron, mg	4.9 ± 2.3	6.0 ± 1.8	7.3 ± 2.0	10.4 ± 5.2	4.9 ± 2.3	6.0 ± 1.8	7.3 ± 2.0	10.4 ± 5.3
Vitamin B12, μg	3.7 ± 3.4	3.8 ± 2.6	4.6 ± 3.4	6.4 ± 6.6	3.7 ± 3.4	3.8 ± 2.6	4.6 ± 3.4	6.4 ± 6.7
Folic acid, μg	184.2 ± 108.6	221.9 ± 80.9	279.2 ± 89.4	434.8 ± 253.9	183.9 ± 108.4	221.3 ± 80.4	278.8 ± 89.9	435.1 ± 257.8
Saturated fatty acids, g	18.7 ± 15.9	16.8 ± 9.0	18.9 ± 9.6	24.3 ± 16.8	18.7 ± 16.0	16.8 ± 8.9	18.9 ± 9.7	24.5 ± 17.0
	**with poor mental HRQOL**				**with poor physical HRQOL**			
	1st quartile	2nd quartile	3rd quartile	4th quartile	1st quartile	2nd quartile	3rd quartile	4th quartile
Energy, kcal	1469.8 ± 761.5	1610.2 ± 453.7	1886.9 ± 516.0	2573.2 ± 1124.2	1462.1 ± 729.8	1603.6 ± 447.4	1854.4 ± 464.3	2442.1 ± 925.3
Protein, g	53.0 ± 36.2	54.6 ± 19.7	63.8 ± 22.8	87.7 ± 45.0	52.9 ± 34.2	54.8 ± 20.1	63.2 ± 20.4	83.4 ± 36.9
Carbohydrates, g	184.8 ± 77.4	217.7 ± 50.6	255.8 ± 55.7	353.1 ± 149.9	184.4 ± 76.6	216.1 ± 47.6	252.2 ± 50.6	337.3 ± 124.9
Calcium, mg	623.4 ± 774.4	456.5 ± 330.1	516.2 ± 360.8	734.2 ± 539.6	603.9 ± 733.2	461.0 ± 331.2	508.7 ± 299.9	701.1 ± 497.8
Iron, mg	5.0 ± 2.5	6.1 ± 1.7	7.4 ± 2.2	10.8 ± 5.4	5.0 ± 2.5	6.1 ± 1.8	7.3 ± 2.0	10.5 ± 4.4
Vitamin B12, μg	3.8 ± 3.7	3.9 ± 2.6	4.7 ± 3.3	6.7 ± 6.7	3.7 ± 3.2	3.9 ± 2.7	4.6 ± 2.8	6.2 ± 4.7
Folic acid, μg	183.8 ± 110.5	221.6 ± 79.8	280.2 ± 96.2	451.9 ± 273.6	187.2 ± 112.5	227.6 ± 85.4	283.7 ± 90.1	447.9 ± 234.8
Saturated fatty acids, g	19.5 ± 16.8	17.4 ± 9.4	20.0 ± 10.9	26.3 ± 17.9	19.2 ± 16.1	17.3 ± 9.7	19.3 ± 9.4	24.5 ± 15.4
	**Unbalanced DP with non-poor mental HRQOL**				**Unbalanced DP with non-poor physical HRQOL**			
Nutrients, *mean* ± *SD*	1st quartile	2nd quartile	3rd quartile	4th quartile	1st quartile	2nd quartile	3rd quartile	4th quartile
Energy, kcal	1961.9 ± 824.4	1689.4 ± 618.8	1687.7 ± 765.9	2574.8 ± 1203.2	1967.6 ± 850.2	1692.9 ± 624.7	1691.5 ± 775.1	1958.0 ± 975.4
Protein, g	69.4 ± 34.7	58.3 ± 25.8	57.1 ± 31.9	94.0 ± 49.4	69.6 ± 35.8	58.5 ± 26.0	57.2 ± 32.2	66.0 ± 39.6
Carbohydrates, g	264.2 ± 104.7	228.2 ± 77.2	230.3 ± 91.8	331.1 ± 159.6	264.8 ± 107.8	228.5 ± 77.8	230.8 ± 93.2	266.8 ± 125.3
Calcium, mg	648.8 ± 499.6	503.3 ± 390.6	491.3 ± 447.1	1146.2 ± 793.3	651.5 ± 506.8	504.8 ± 394.2	493.0 ± 449.2	605.9 ± 649.4
Iron, mg	8.7 ± 4.3	6.5 ± 2.6	6.1 ± 3.5	9.7 ± 5.8	8.7 ± 4.4	6.5 ± 2.6	6.1 ± 3.5	7.2 ± 3.8
Vitamin B12, μg	5.3 ± 4.8	4.3 ± 3.2	4.1 ± 3.7	7.2 ± 7.1	5.3 ± 5.1	4.3 ± 3.3	4.1 ± 3.7	4.8 ± 5.4
Folic acid, μg	380.4 ± 221.5	244.4 ± 111.8	217.8 ± 141.7	413.4 ± 290.9	381.8 ± 228.1	244.8 ± 112.4	218.2 ± 142.5	274.4 ± 170.7
Saturated fatty acids, g	20.7 ± 13.1	18.1 ± 10.7	18.2 ± 12.7	32.9 ± 20.5	20.8 ± 13.4	18.2 ± 10.8	18.2 ± 12.7	21.7 ± 16.8
	**with poor mental HRQOL**				**with poor physical HRQOL**			
	1st quartile	2nd quartile	3rd quartile	4th quartile	1st quartile	2nd quartile	3rd quartile	4th quartile
Energy, kcal	2109.3 ± 1100.3	1764.4 ± 693.1	1734.2 ± 751.1	2025.3 ± 919.2	2018.3 ± 829.9	1712.5 ± 622.5	1689.5 ± 640.6	1978.9 ± 891.0
Protein, g	75.6 ± 45.6	60.9 ± 27.5	58.7 ± 29.6	67.7 ± 36.9	71.8 ± 33.6	59.0 ± 25.0	57.2 ± 26.7	66.9 ± 36.1
Carbohydrates, g	278.3 ± 139.1	235.4 ± 84.9	235.1 ± 95.1	275.3 ± 125.3	269.3 ± 106.1	230.8 ± 78.1	229.2 ± 78.3	267.6 ± 121.6
Calcium, mg	702.5 ± 575.9	524.5 ± 407.1	509.1 ± 451.9	617.1 ± 648.2	664.0 ± 495.7	504.4 ± 365.0	489.7 ± 429.0	605.2 ± 641.8
Iron, mg	9.5 ± 5.7	6.7 ± 2.8	6.3 ± 3.0	7.4 ± 3.7	9.0 ± 4.1	6.6 ± 2.6	6.1 ± 2.7	7.4 ± 3.9
Vitamin B12, μg	5.9 ± 7.0	4.5 ± 3.2	4.2 ± 3.2	4.9 ± 4.1	5.4 ± 4.3	4.3 ± 3.0	4.0 ± 2.8	4.7 ± 3.8
Folic acid, μg	416.6 ± 287.9	254.1 ± 119.0	223.1 ± 121.8	279.7 ± 164.8	394.4 ± 218.2	248.3 ± 111.3	218.2 ± 111.8	284.6 ± 175.6
Saturated fatty acids, g	22.9 ± 17.3	19.4 ± 11.9	18.9 ± 12.4	22.6 ± 16.1	21.6 ± 14.1	18.5 ± 10.7	18.5 ± 11.7	21.9 ± 15.5
